# Abscess of the Gall-Bladder—Rupture and Evacuation into the Peritoneal Cavity—Death—Autopsy

**Published:** 1864-04

**Authors:** Henry M. Lyman

**Affiliations:** Chicago


ARTICLE XV.

### ABSCESS OF THE GALL-BLADDER—RUPTURE AND EVACUATION INTO THE PERITONEAL CAVITY—DEATH—AUTOPSY.

By HENRY M. LYMAN, of Chicago.

Frederic Meuser, private in the 19th Reg't Illinois Vol. Infantry, aged 28, was admitted to the University Hospital, Nashville, Tenn., April 26, 1862, at which time he was supposed to be suffering with typhoid fever. He had been since the 19th of January, 1862, an inmate of various military hospitals in Kentucky and Tennessee, where he had experienced a succession of icteric attacks, which had permanently tinged his skin a deep tawny hue. He would complain of severe pain in the epigastric region; this would be followed by irritative fever, continuing for a few days, during which time the skin and conjunctivæ assumed a bright yellow tint. These symptoms would then gradually subside, leaving only the tawny complexion and the dull epigastric pain to indicate the persistence of disease. In this way he lingered, a great puzzle to his surgeon, till the 20th of June, 1862, when he was transferred to my care. At this date the patient declared himself comparatively free from pain; he was able to leave his room, and to walk in the open air for a short time each day. Soon, however, he again began to complain of incessant pain behind the xiphoid cartilage, and a sensation of weight and fulness in the right hypochondrium. The skin resumed its icteric hue; the urine became high-colored; the stools were loose, watery, and bright-colored; the tongue was colored with short, white, moist fur; there was a succession of febrile paroxysms, alternating with periods of profuse perspiration, simulating by their regularity, a quotidian intermittent, the fever (usually without any previous chill) commencing a little before noon, and continuing till twilight, when the sweating stage would begin, and frequently persist till daylight the following morning. The region of hepatic dulness extended as high as the nipple, and below the costal cartilages the border of the liver projected two inches towards the umbilicus. The epigastric region was extremely sensitive to pressure.

The treatment which seemed to afford the greatest relief, consisted in the application of cups, sinapisms, turpentine stuper, and flaxseed poultices over the abdomen, accompanied by the administration of alkalies with colocynth. Quinine was ordered, but was soon discarded as ineffectual in controlling the febrile paroxysms, which were then proved to be non-malarial in their character.

The saffron-hued complexion of the patient soon gave place to a singular paleness which, however, did not wholly obliterate the ingrained tawny color of the skin. The febrile irritation of the system was also somewhat abated. On the 5th of July, the patient announced a complete translation of pain from the epigastrium to the region of the sigmoid flexure of the colon. He now preferred to lie upon his right side, and seemed to be relieved of a great deal of suffering. The right side of the abdomen was painless and dull on percussion, while the left side was everywhere resonant and sensitive to pressure. On the following day, but one, the deep-seated pain was restored to its original location, and the right side of the abdomen gave forth its usual resonance. The patient contiuued to lie upon his right side.

From this time the region of the epigastrium became tense, and gradually distended. There were no more paroxysms of fever—only a dull and constant sense of distress, which required the frequent administration of morphine, to mitigate its severity.

Thus lingered our patient till the middle of July, when he experienced a sudden cessation of the pain which had tormented him. The whole abdomen now began to enlarge, till it seemed as if its walls could endure no farther expansion. There was considerable emaciation; the sufferer rested always upon his right side, and complained only of uneasiness and distress that pervaded the entire abdomen. The pulse was small, soft, and scarcely accelerated; the tongue retained, without change, the appearance which has already been described; the skin was dry but not harsh; the feet were somewhat swelled; the depending portion of the abdomen was dull on percussion, while its inferior portion was decidedly tympanitic.

At 6 o'clock, P.M., July 23d, the patient desired to relieve his bowels. He assisted himself to the stool, and after defecation returned to his couch, remarking, with a smile, that his attendant was not strong enough to help him. He then placed himself quietly in bed, when he gasped once or twice, and was dead.

*Autopsy fifteen hours after death.*—The head was not inspected. *Thorax.*—The apex of the right lung was adherent to the costal pleura. It contained several small calcareous masses, surrounded by a zone of chronic pneumonia. The whole of the left lung was bound by old adhesion to the costal pleura, and the posterior portion of its apex was puckered and contracted over a mass of chronic pneumonia as large as a lemon. The valves of the heart were healthy; the right ventricle contained a large colorless clot; the external surface of the organ was loaded with fat. The pericardium contained two ounces of serous fluid. The right pleural cavity also contained nearly a pint of similar fluid.

*Abdomen.*—The skin and superficial fascia were carefully incised from the xiphoid cartilage to the pubes. The point of a scalpel was then pushed through the fibrous aponeurosis near its attachment to the ensiform cartilage, and a jet of horribly offensive, chocolate-colored pus followed the withdrawal of the blade. A bloody fluid, unmixed with pus, escaped as the knife approached the umbilicus; and when its edge had reached the symphysis pubis, the table was deluged with pus, blood, and a clear serous fluid, which had occupied the hypogastric cavity, measuring, altogether, three or four ounces over two gallons. The intestines were void of solid contents, and were distended with gas; their peritoneal surface was a lustreless white, sprinkled with small dark spots, giving it much the appearance of mildewed linen. The intestinal coils were slightly adherent, united by coagulated fibrin which could be easily separated from the peritoneum. The pelvic cavity and the hollow of the right flank were filled with white clots like those which are often found in the heart after death. Passing the hand below the liver, a large number of gall-stones were discovered within the the peritoneal cavity, between the liver and the caput coli. The gall-bladder was utterly destroyed, only a small portion of the cystic duct remained. The hepatic duct and the common duct were entire, and contained yellow bile. The diameter of the common duct varied little from that of a goose-quill. The left lobe of the liver adhered in such a way to the anterior surface of the stomach, intestines, and anterior wall of the abdomen, that a continuous cavity extended from the gate of the liver to its anterior border, where the fundus of the gall-badder had been situated, then passing over the upper surface of the left lobe of the liver, to the left of the falciform ligament, and so on between the liver and the diaphragm to the spinal column. This was the cavity which had yielded pus and dark clots of blood. The gastric wall of this abscess was permanently stained with blood, which appeared to have issued from a twig of the cystic artery. The mucous surface of the stomach and intestinal canal presented no unusual appearance. The spleen was of a dark, chocolate color; it was not enlarged, and its texture was firm. The kidneys, too, were scarcely diseased; their size was normal, and it was only by a yellowish discoloration of the cortical portion that they differed from the natural appearance of those organs. The liver was not enlarged, but its whole substance was of an olive-green color, and the lobules were centrally congested. The pancreas was firm and normal in its appearance. When removed from the body, the gall-stones filled a two-ounce vial; they varied in size from a mustard seed to a small olive.

*Remarks.*—This case presents an excellent illustration of the consequences of biliary concretions enlarging and multiplying within the gall-bladder. It is probable that gall-stones were formed during the early stages of the disease, which confined the sufferer to the wards of the military hospitals. The youth of the patient favors this hypothesis, for it is a rare thing to find gall-stones in a person under thirty years of age. Undoubtedly the hepatic disturbances resulting from the typho-malarial influences to which this man had been exposed, aggravated, if they did not actually originate the tendency which declared itself in so alarming a form.

It would seem that none of the larger calculi were ever forced through the biliary ducts, for the common choladoch duct retained its normal calibre, and the patient never experienced those agonizing colics which are supposed to indicate the passage of a large or angular gall-stone into the duodenum. The symptoms in this case were those which would indicate the passage of calculi which, though large enough to produce jaundice by occlusion of the common duct, were too small to excite severe pain by forcible dilatation of that canal. The final catastrophe, however, must have been provoked by the detention of a large stone in the cystic duct, where it may have excited the ulceration that destroyed the whole viscus, and discharged its contents into the peritoneal cavity. The reparative efforts of nature are well shown by the limits so speedily thrown about the various effusions: the rupture of the gall-bladder was attended by hemorrhage, which filled the right flank and the pelvic cavity with blood. The speedy coagulation of this effusion retained the gall-stones in that portion of the abdomen which they reached, as they fell from their nidus. The fibrinous adhesions between the coils of the intestines confined the clotted masses which soon parted with their coloring matter. The bloody serum, in like manner, was so walled in that it never mixed with that transparent fluid, the most recent of all the exudations, which occupied the greater portion of the abdomen, until the peritoneal cavity had been evacuated, and its contents were mingled upon the dissecting table. Surrounded by inflammatory adhesions, the purulent effusion thrown off by the ulcerated hepatic surface (all that was left of the gall-bladder,) was steadily pushing itself upwards and outwards. Had the patient lived a few days longer the abscess might have perforated the diaphragm, or it might have pointed externally. The engorgement and discoloration of the liver seems to have resulted from the pressure to which the viscus was latterly subjected, impeding the sanguineous circulation, rather than from any specific local obstruction of the biliary flow. The insignificance of the symptoms which marked the gravest accidents is also worthy of note. The rupture of the gall-bladder occasioned nothing to excite alarm. It was attended with relief, rather than increase of pain. From that time the suffering of the patient seemed to grow out of the inconveniences produced by excessive abdominal distention; they were at no time characteristic of acute peritonitis, such as occurs after perforation of the gastro-intestinal canal, or rupture of the uterus. Death was the result of exhaustion, or, as some would have us believe, it was necessitated by the circumstances which caused the formation of a white clot in the right venticle of the heart.

